# Nano-Selenium Alleviates Cadmium-Induced Acute Hepatic Toxicity by Decreasing Oxidative Stress and Activating the Nrf2 Pathway in Male Kunming Mice

**DOI:** 10.3389/fvets.2022.942189

**Published:** 2022-07-26

**Authors:** Hong Du, Yilei Zheng, Wei Zhang, Huaqiao Tang, Bo Jing, Haohuan Li, Funeng Xu, Juchun Lin, Hualin Fu, Lijen Chang, Gang Shu

**Affiliations:** ^1^Department of Basic Veterinary Medicine, Sichuan Agricultural University, Chengdu, China; ^2^College of Veterinary Medicine, University of Minnesota, St Paul, MN, United States; ^3^Department of Veterinary Clinical Science, College of Veterinary Medicine, Oklahoma State University, Stillwater, OK, United States

**Keywords:** cadmium, nano-selenium, liver, oxidative stress, Nrf2

## Abstract

Cadmium (Cd) is known as a highly toxic heavy metal and has been reported to induce hepatotoxicity in animals. Nano-selenium (NSe) is an antioxidant that plays many biological roles such as oxidative stress alleviation. The purpose of this study is to explore the mechanism of action by which NSe inhibits Cd-induced hepatic toxicity and oxidative stress. Sixty eight-week-old male Kunming mice were randomly divided into four groups (15 mice per group). The control group and cadmium groups received distilled water, whereas the sodium-selenite group received 0.2 mg/kg SSe and the NSe group received 0.2 mg/kg NSe intragastrically for 2 weeks. On the last day, all the other groups were treated with Cd (126 mg/kg) except for the control group. The results obtained in this study showed that NSe alleviated Cd-induced hepatic pathological changes. Furthermore, NSe reduced the activities of ALT and AST as well as the content of MDA, while elevated the activities of T-AOC, T-SOD and GSH (*P* < 0.05). In addition, the NSe group significantly increased mRNA expressions of Nrf2 pathway related molecules (Nrf2, HO-1, NQO-1, GST, GSH-Px, CAT and SOD) compared to the Cd group (*P* < 0.05). In conclusion, NSe shows its potentiality to reduce Cd-induced liver injury by inhibiting oxidative stress and activating the Nrf2 pathway.

## Introduction

Cadmium (Cd) is a heavy metal characterized with silvery-white color and ductile texture. It is commonly used in the manufacture of protective plates, batteries and pigments because of its characteristics such as water insoluble, non-flammability, and corrosion-resistant (1, 2). The imbalance between Cd consumption and recycling of Cd compounds has become a challenge (3). In China, reports indicated that approximately 2.8 × 10^5^ hectares of farmland are polluted by Cd (4). It has been reported that Cd contaminated sewage could be absorbed by plants but Cd cannot be chemically biodegraded by microorganisms (5–7). Furthermore, the half-life of Cd is several decades which makes it accumulates in human and animals tissues easily throughout their life-time (8, 9). Cd accumulation in animals and humans cause malfunctioning of multiple organs and tissues such as liver, kidneys, testicles, lungs, bone, spleen as well as nervous system. The extent of Cd damage depends on the route, dose and exposure time (10–12). Currently Cd is recognized as a potentially hazardous chemical to global public health and agricultural food security. Therefore, exploring effective methods to prevent and treat Cd-induced toxicity in animals and humans is essential.

The accumulation of Cd in the body negatively affects liver function by forming of reactive oxygen species (ROS), leading to oxidative stress (13, 14), which has been considered as the major mechanism of Cd-induced hepatic toxicity (15, 16). The nuclear factor erythrocyte 2-related factor 2 (Nrf2) is activated during oxidative stress and interacts with the antioxidant response element (ARE) to upregulate the transcription of antioxidant genes and detoxification enzymes (17–19). The genes and enzymes include NAD(P)H quinone oxidoreductase 1 (NQO1), heme oxygenase-1 (HO-1), catalase (CAT), and glutathione peroxidase(GSH-Px) and so on (20). It was proposed that the activation of the Nrf2 signaling pathway is of vital importance against Cd-induced liver damage because it prevents Cd-induced oxidative stress and liver toxicity (16, 21–23).

Selenium (Se) is a naturally occurring metalloid element with various natural forms and is an essential trace element for humans, plants, and animals (23). Se shows its unique capability as an antioxidant *via* scavenging free radicals (24, 25). Se had an antagonistic effect toward mercury, cadmium, lead and many other substances (26). Compared with the sodium selenite (SSe), nano-selenium (NSe) has better biological effects, antioxidant ability and lower toxicity (24). Se antagonizes Cd by increasing the glutathione levels to minimize oxidative stress, therefore alleviating Cd-induced damage (26–28). Furthermore, it has been proven that Se upregulates the expression of Nrf2-dependent antioxidant enzymes which enhances its protection against specific pathological changes (28–30).

It has been reported that NSe alleviates Cd-induced hepatotoxicity (23, 25). However, the mechanism by which this alleviation occurs remains unclear. Therefore, the purpose of this study is to explore whether NSe protects liver from Cd-induced toxicity *via* Nrf2-mediated pathway. The results of this study provides information for further understanding of the protective mechanism of NSe against cadmium poisoning.

## Materials and Methods

### Chemicals

Cadmium chloride (CdCl_2_), sodium-selenite (Na_2_SeO_3_) and sodium pentobarbital (C_11_H_17_N_2_NaO_3_) were bought from Chengdu Kelong Chemical Co., Ltd (Chengdu, China). Nano-selenium, used during current study was acquired from the Physical Chemistry Laboratory of Sichuan Agricultural University, China. Reagent kits for the determination of biochemical parameters (alanine aminotransferase (ALT), A305-1; aspartate aminotransferase (AST), A409-1; MDA, A203-1; glutathione (GSH), A216-2; and total superoxide dis-mutase (T-SOD), A201-1) were purchased from the Nanjing Jiancheng Bioengineering Institute of China (Nanjing, China). PrimeScript™ RT Reagent Kit (cat no. RR086A), SYBR® Premix Ex Taq™ II (cat no. RR920A), and RNAiso Plus (cat no. 9178) were supplied by Takara Biotechnology Co., Ltd. (Dalian, Liaoning, China).

### Animals and Treatments

The use of laboratory animals was approved by the Animal Care and Use Committee of Sichuan Agricultural University (Approval number DYY-2020203012). Sixty male Kunming mice (eight weeks old, 18–22g) were purchased from Dashuo Biological Technology (Chengdu, China, Animal license number: SCXK (chuang) 2020-030). All the mice were kept in a standard SPF facility at room temperature of 25 ± 2°C, and were subjected to a 12-h light-dark cycle and fed with the standard pellet diet with free access to water. To investigate the protective effect of NSe on Cd-induced acute liver injury, sixty mice were randomly divided into four groups (15 mice per group) after 1 week of adaption: (1) control group (CON); (2) cadmium group (Cd); (3) nano-selenium group (NSe); (4) sodium-selenite group (SSe). Nano-selenium (0.2 mg/kg/day) and sodium-selenite (0.2 mg/kg/day) were dissolved in distilled water and were administered *via* a gastric tube to mice for 14 days. The mice in the control group and cadmium groups were treated with an equal volume of distilled water. The doses of NSe and SSe were determined following the procedures described previously (31). After the last administration of treatments, the mice in the nano-selenium group; sodium-selenite group; and cadmium group were given Cd dissolved in distilled water (126 mg/kg of body weight) (32), the control group were given equal volume of the distilled water, gavage administration. After 24 hours of Cd challenge, animals were anesthetized with 80 mg/kg of 2% sodium pentobarbital administered by intraperitoneal injection, followed by dislocation of cervical vertebrae. Blood was taken from orbital vein of mice. Serum alanine aminotransferase (ALT), aspartate aminotransferase (AST) and biomarkers of oxidative stress were assessed. The liver samples were collected, weighed and stored for histopathological and biochemical assessments including biomarkers of oxidative stress and gene expression.

### Body Weight, Liver Weight and Liver Index

The body weight of the mice was measured and recorded weekly. Liver samples were collected, weighed, and photographed at the end of experiment. The liver index was determined by the formula below:


(1) $$\begin{array}{r} Liver\;index\;\left(\%\right)=\frac{liver\;weight\left(mg\right)}{Body\;weight\left(g\right)}\;\times 100\% \end{array}$$

### Activity of Serum ALT and AST

Blood samples from the mice were centrifuged at 3,000 rpm for 10 min. Commercial kits were used to measure the serum levels of alanine aminotransferase (ALT) and aspartate aminotransferase (AST). The measurement procedure was performed according to the manufacturer's instructions (Nanjing Jiancheng Bioengineering Institute of China, Nanjing, China).

### Detection of Serum Oxidative Stress Indicators in Serum

Blood samples were taken from the mice at the end of the experiment. Serum was obtained after being centrifuged at 3,000 rpm for 10 min. Activities of the total superoxide dismutase (T-SOD), total antioxidant capacity (T-AOC), and the concentrations of glutathione (GSH) and malondialdehyde (MDA) were measured by specific biochemical reagent kits provided by Nanjing Jiancheng Bioengineering Institute of China (Nanjing, China).

### Assessment of Oxidative Stress Indicators in the Liver

Liver samples were removed, washed in 4°C saline solution, weighed and homogenized in an ice-cold 0.9%NaCl solution at a ratio of 1:9 (w/v) followed by centrifugation (at 3,500 rpm for 10 min at 4°C). Then the suspension was collected for determination of total protein by use of the Bradford protein assay. The activities of T-SOD and T-AOC, and the concentration of GSH and MDA in hepatocytes were detected by the aforementioned reagent kits (Nanjing, China).

### Histopathological Evaluation

Liver samples were collected, fixed in 4% paraformaldehyde, dehydrated in alcohol series of ethanol solutions, and embedded by paraffin. Samples were sliced into 4 μm thickness and stained by hematoxylin and eosin (HE) for assessment of microscopic lesions (33).

### RNA Extraction and Quantitative Real-Time PCR (qRT-PCR)

The livers samples were stored at −80°C for RNA extraction. Hepatic total RNA was extracted by RNAiso Plus (9108, Takara, Otsu, Japan) following the manufacture's instructions. cDNA was synthesized using one microgram of RNA concurrently with the Prim-ScriptTM RT reagent Kit (9108, Takara, Japan) and SYBR Premix Ex TaqTM II kit (9108A, Takara, Japan). The RNA expressions, including Nrf2, HO-1, GST, GSH-Px, SOD1, CAT, and NQO-1, were quantified by Real-Time quantitative PCR (Bio-Rad, USA). β-actin was used as the house-keeping gene to normalize the gene expression, and the 2-^ΔΔCT^ method was used for calculation of the RNA expression levels (34). The primers (Table 1) were designed by using of Premier 5 (PREMIER Biosoft International, Palo Alto, CA) and synthesized by Qingke BioTech Co., Ltd (33).

**Table 1:** Primers used in qRT-PCR.

**Gene**	**Accession number**	**Primer sequence (5^**′**^-3^**′**^)**	**Product length(bp)**
*Nrf2*	NM-010902.4	F: AGCGGTAGTATCAGCCA R: GCCCAGTCCCTCAATAGC	150
*HO-1*	NM-010442.2	F: TGTTGCGCTCTATCTCC R: GTACACATCCAAGCCGAG	136
*GST*	NM-019946.5	F: AAGATTCTGAAGTGCAT R: AAGTTTGTTCGCACTGA	149
*GSH-Px*	NM-008160.6	F: TCTCTTCATTCTTGCCAT R: GACTACACCGAGATGAACGA	112
*SOD1*	NM-011434.2	F: CACCTTTGCCCAAGTCA R: TCCATTGAAGATCGTGT	97
*CAT*	NM-009804.2	F: TTCCTGAGCAAGCCTTC R: CACATGAATGGCTATGGATCACA	138
*NQO-1*	NM-036153810.1	F: GGCCAATGCTGTAAACCA R: GCGGCTCCATGTACTCT	129
*β-actin*	NM-007393	F: CGCTCGTTGCCAATAGTG R: GCTGTGCTATGTTGCTCTAG	117

### Statistical Analysis

All the data were analyzed by SPSS 27.0 (SPSS Inc., Chicago, IL, USA). One-way analysis of variance (ANOVA) was used to determine the overall difference among all groups. All parameters were presented as mean ±standard deviation. Significant difference was defined when *P* < 0.05.

## Results

### Liver Weight and Index

**Figure 1:**
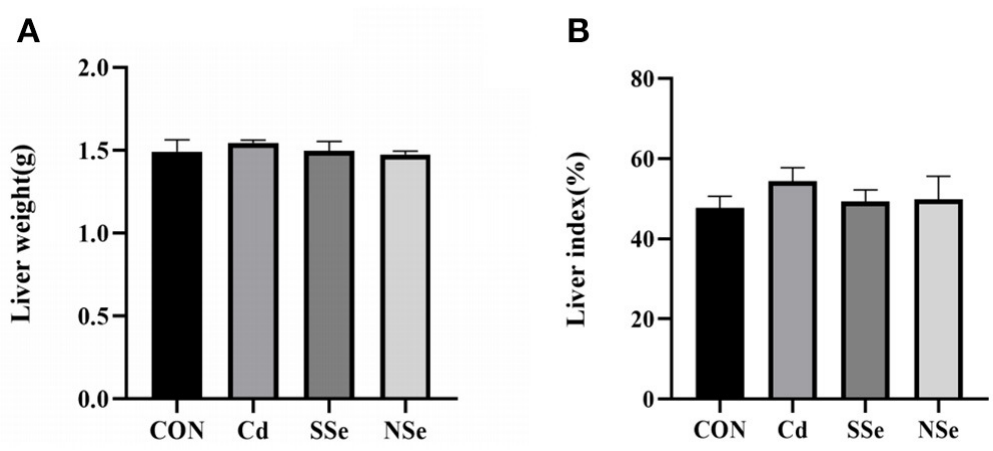
Hepatic weight and liver index. **(A)** Liver weight; **(B)** Liver index. Data were presented as the mean ± SD. Different letters represented a statistically significant difference (*P* < 0.05) within the column, and the same letters represent no significant difference (*P* > 0.05). CON, control group; Cd, cadmuim group; SSe, sodium-selenite group; NSe, nano-selenium group.

As shown in Figure 1A, there was no significant difference observed in the liver weight of the mice. Moreover, no significant alterations of the liver index were shown among the four groups in Figure 1B (*P* > 0.05). Although no significant difference was detected, the hepatic index in the Cd group increased slightly compared to that of other groups.

### Activities of ALT and AST in Serum

**Figure 2:**
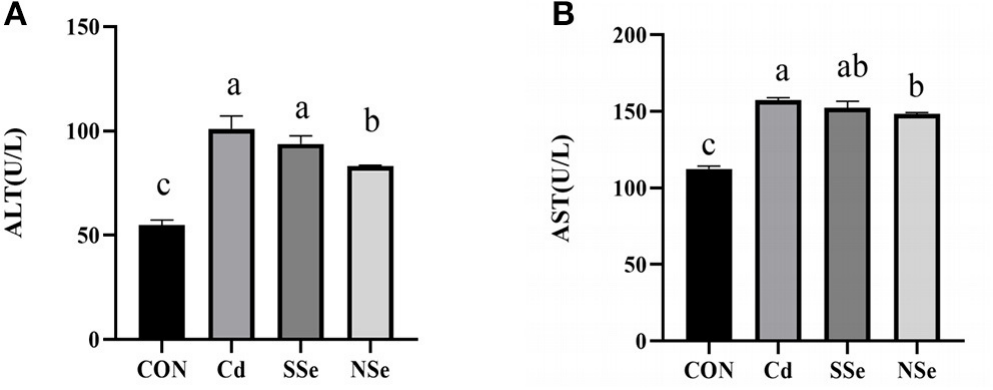
Activities of ALT and AST in serum. **(A)** Serum ALT level; **(B)** Serum AST level. Different letters represent significant difference (*P* < 0.05) within the column, and the same letters represent no significant difference (*P* > 0.05).

The levels of serum ALT and AST in treatment groups increased significantly when compared to control mice (*P* < 0.05). However, the serum activities of ALT and AST were significantly lower in the NSe group compared to the Cd group (*P* < 0.05), but remained significantly higher than the control group (*P* < 0.05). No significant changes of ALT and AST were noticed between the Cd group and SSe group in Figure 2.

### Activities of Antioxidant Enzymes and Concentrations of Oxidative Stress Indicators in Serum

**Figure 3:**
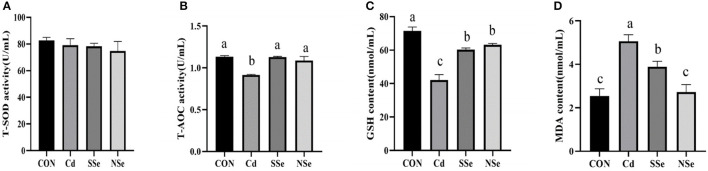
Activities of antioxidant enzymes and concentrations of oxidative in serum. **(A)** T-SOD; **(B)** T-AOC; **(C)** GSH; **(D)** MDA. Different letters represent significant difference (*P* < 0.05) within the column, and the same letters represent no significant difference (*P* > 0.05).

The results of serum levels of the antioxidative enzymes are shown in Figure 3. The serum activities of T-AOC and GSH decreased significantly in the Cd group, whereas the content of MDA in the Cd group elevated significantly compared to the control group (*P* < 0.05). The serum level of GSH decreased significantly in NSe and SSe groups compared to the control group (*P* < 0.05). Additionally, the serum content of MDA declined significantly in the NSe group compared to the Cd and SSe groups (*P* < 0.05). However, no significant differences were detected in the serum activities of T-SOD among all groups (*P* > 0.05).

### Activities of Antioxidant Enzymes and Concentrations of Oxidative Stress Indicators in Liver

**Figure 4:**
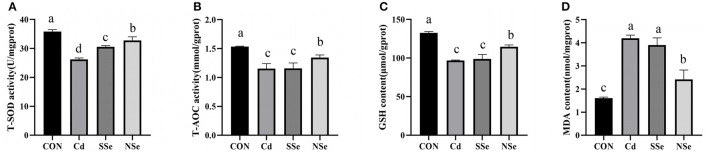
Activities of antioxidant enzymes and concentrations of oxidative in liver. **(A)** T-SOD; **(B)** T-AOC; **(C)** GSH; **(D)** MDA. Different letters represent significant difference (*P* < 0.05) within the column, and the same letters represent no significant difference (*P* > 0.05).

The results of hepatic levels of oxidative stress indicators are shown in Figure 4. The activities of hepatic T-SOD, T-AOC, and GSH decreased significantly in the Cd group compared to the control group (*P* < 0.05). The hepatic T-SOD, T-AOC, and GSH activities elevated significantly in the NSe group compared to the Cd group (*P* < 0.05), whereas the content of MDA decreased significantly between these two groups. The level of hepatic T-SOD in the SSe group elevated significantly compared to the Cd group, the level was significantly lower than the NSe group (*P* < 0.05).

### Hepatic Morphology

**Figure 5:**
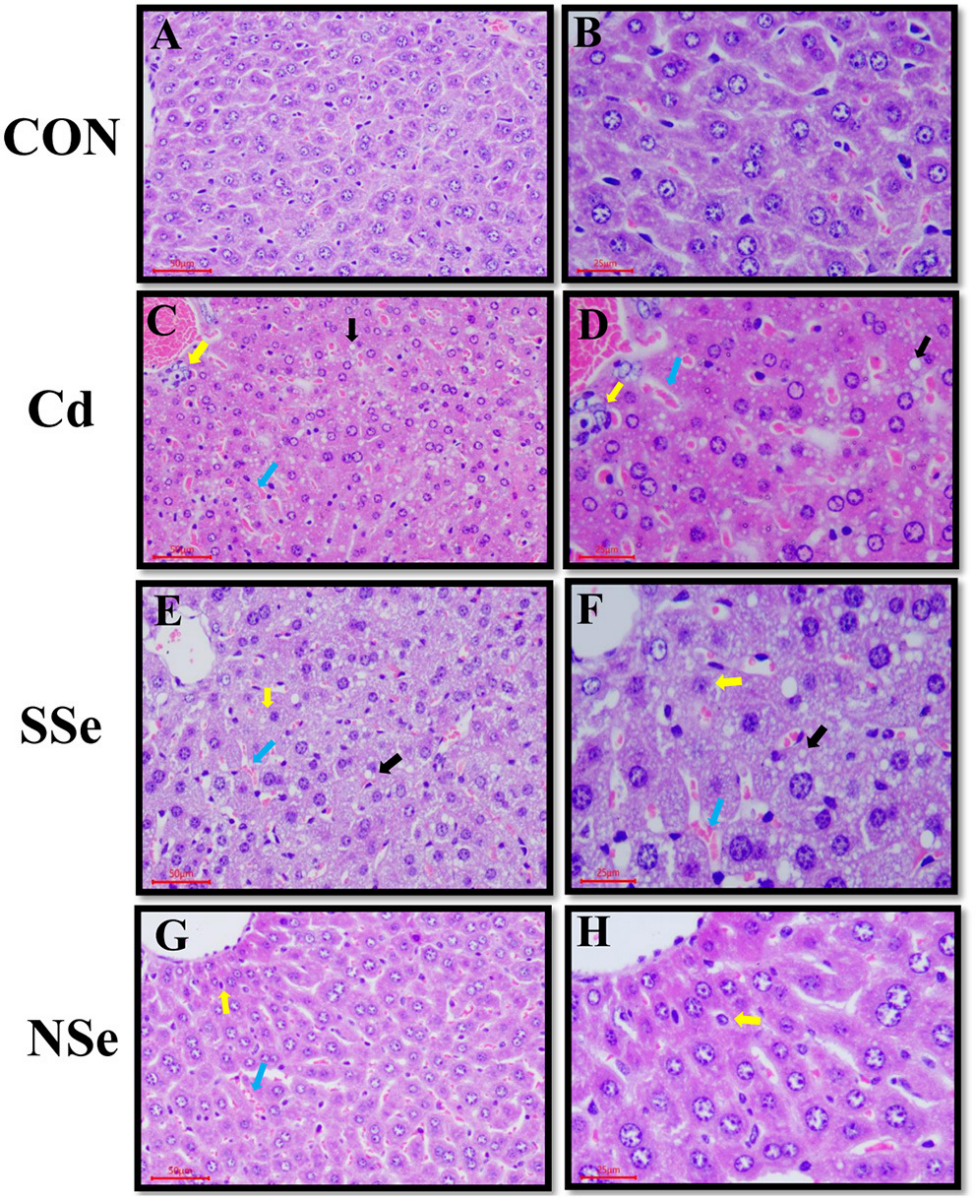
Hepatic morphology, the left magnification:50 μm, 200X; the right magnification:25 μm, 400X. Yellow arrows: necrotic hepatocytes; black arrows: fatty degeneration; bule arrows: red blood cells. **(A,B)** Represent changes in the liver of control group. **(C,D)** Represent changes in the liver of cadmium group. **(E,F)** Represent changes in the liver of sodium-selenite group. **(G,H)** Represent changes in the liver of nano-selenium group.

Histopathological changes in the liver are shown in Figure 5. Hepatocytes of the control group had normal structures with regular hepatic cords, portal triad, and intact hepatocytes (Figures 5A,B). In the Cd group, the histopathological changes showed the unclear border of hepatic lobules, irregular arrangement of hepatic cords with fatty changes, necrosis, and congestion (Figures 5C,D), which suggested that Cd-induced hepatic injuries in the mice. Compared with the Cd group, the histopathological lesions in the NSe group were less significant subjectively compared to the Cd group (Figures 5G,H), whereas there were clear cytological changes observed in the SSe group (Figures 5E,F).

### Relative mRNA Expressions of the Nrf2 Pathway

**Figure 6:**
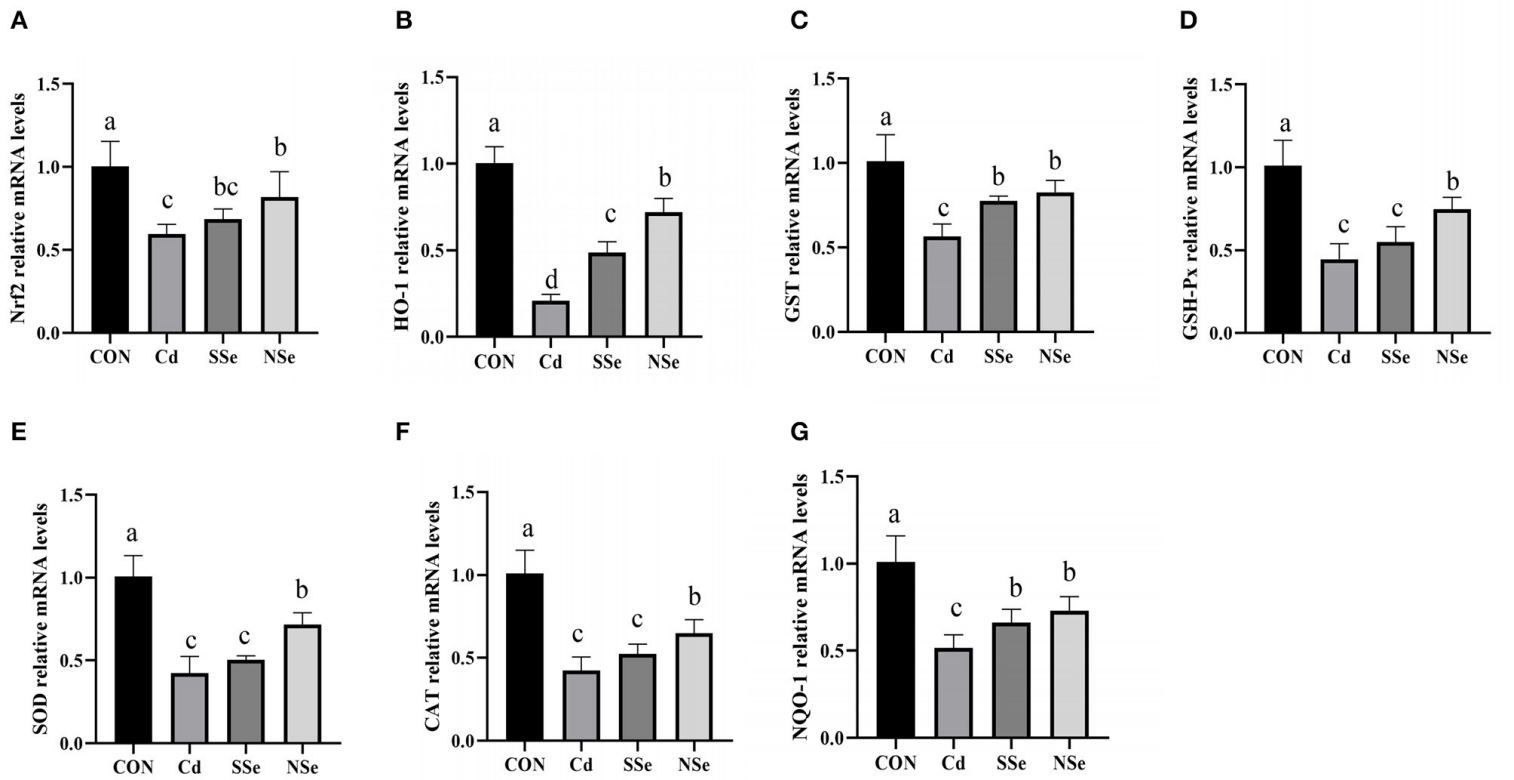
The relative mRNA expression levels of Nrf2, HO-1, GST, GSH-Px, SOD, CAT and NQO-1 in the liver for this experiment. **(A)** Nrf2, **(B)** HO-1, **(C)** GST, **(D)** GSH-Px, **(E)** SOD, **(F)** CAT, **(G)** NQO-1. Different letters represented significant difference (*P* < 0.05).

The effects of Nano-selenium on expressions of the Nrf2 pathway mRNA are shown in Figure 6. The mRNA expressions of Nrf2-related molecules (Nrf2, HO-1, NQO-1, GSH-Px, CAT, SOD and GST) in hepatocytes declined significantly in the Cd group compared with the control group (*P* < 0.05). The expressions of aforementioned mRNA were significantly higher in the NSe group compared to the Cd group (*P* < 0.05), yet were still lower than the control group (*P* < 0.05).

## Discussion

Cadmium (Cd) has been recognized as a toxic heavy metal which accumulates in the liver of both humans and animals (14, 34, 35). Induction of reactive oxygen species (ROS) is one of the recognized reasons that Cd causes hepatotoxicity. Excessive ROS produced by oxidative stress leads to lipid peroxidation and breakdown of DNA strands (1, 10, 36). As an antioxidant agent, NSe plays a crucial role in the reduction of free radicals to protect cells from damage by regulating reactive oxygen species (ROS) and glutathione peroxidases (GPx) (23, 37, 38). Although there is some evidence that NSe had hepatoprotective and antioxidant property (23), whether NSe regulates Nrf2-mediated pathway against Cd-induced liver injury has not been reported. Therefore, in the present study, we explore regulatory roles of NSe toward Nrf2 pathway to protect the liver against oxidative damage induced by Cd.

Oxidative stress is reported to play a crucial role in Cd-induced hepatotoxicity. Superoxide, nitric oxide, and hydroxyl radicals are produced when hepatocytes are exposed to Cd. The stress generated leads to instability of the cell membrane followed by disintegration (36, 39). In the present study, both serum AST and ALT increased after Cd administration, indicating that Cd induces hepatic injuries, since concurrent elevation of serum AST and ALT levels are considered a cardinal sign to diagnose reduction of liver function or hepatocyte damage (14). The balance between the production of ROS and the antioxidant mechanism is lost when excessive ROS is produced. SOD, GSH and T-AOC could drop, whereas the MDA level rises under oxidative stress (33). SOD represents the activity of antioxidant enzymes, and eliminates excessive ROS (14). MDA levels is an important parameter indicating the increase in lipid peroxidation rate (36). Glutathione (GSH) is an antioxidant synthesized in cells with low molecular weight, and can reduce the generation of free radicals (35), whereas the T-AOC represents the total antioxidant capacity of the body (33). The results of the present study showed that compared with the control group, administration of Cd alone increased the activities of serum AST and ALT, while decreased the activities of SOD, and the levels of T-AOC and GSH in the liver. The contents of MDA in serum increased while the levels of T-AOC and GSH dropped, compared with the control group. These results indicate that Cd could cause liver damage and oxidative stress, which corresponds with other reports (13, 14, 19). However, treatment with NSe could significantly reduce concentrations of serum AST and ALT, MDA, while increase the activities of SOD, and the levels of T-AOC and GSH in the liver, compared with the Cd group. These results substantiated that NSe could reverse the Cd-induced hepatic oxidative stress and damage, which corresponds with other reports (23, 25). Importantly, compared with the SSe group, treatment with of NSe could significantly reduce concentrations of serum ALT and MDA, while increasing concentration of SOD, GSH and T-AOC. These results suggest that NSe is better at resisting Cd-induce liver damage than SSe.

The histopathological examination helps to evaluate organ structural damage. Our study showed that the Cd group had unclear hepatic lobules, disorganized hepatocyte cords, some fatty degeneration, and necrosis in hepatocytes. These results indicate that Cd could cause obvious pathological injury, which is consistent with previous studies (33, 40). The fatty degeneration, necrosis, and congestion of hepatocytes were the main histopathological damage induced by Cd, indicating that Cd exposure may cause hepatocyte interior injury. However, it is worth noting that the different histological changes of liver caused by Cd were also reported. For example, Cd could induce severe granular and vacuolar degeneration after gavaging of CdCl_2_ in the male Sprague-Dawley rats (5 mg/kg/day) for 28 days (33). After receiving of CdCl_2_ by gavage (5 mg/kg/day) for 30 days, Kunming mice had ballooning degeneration and inflammatory cell infiltration in the liver (41). Although obvious vacuolar degeneration has not been observed in this study, it is more likely due to the different time, dosage, route and experimental condition of Cd exposure conducted in this study.

Nuclear factor erythroid 2-related factor 2 (Nrf2) is a transcription factor that upregulates protective genes in response to oxidative stress (21, 22). It is located in the cytoplasm and combined with Keap1, a sensor of oxidative stress, for proteasomal degradation under physiological conditions (20, 21). Nevertheless, after being released from Keap1, it translocates into the nucleus and binds to the antioxidant response element (ARE) in response to oxidative stress (22). Nrf2 target genes include NAD(P)H quinone oxidoreductase 1 (NQO1), GSH conjugation (Gsts), and many other oxidized protein repair related genes. Activation of the Nrf2 pathway increases the expression of these genes (33). Our study demonstrates that Cd inhibits the activation of the Nrf2 signaling pathway at the molecular level by significantly down-regulating the mRNA and protein expressions of Nrf2, as well as its downstream factor HO-1, NQO-1, and GST in the liver tissue. Findings from the vast majority of the studies support the notion that Cd exposure restrains Nrf2.

For example, Bashir et al. explained that Cd exposure after a 4-week intragastric administration of CdCl_2_ (5 mg/kg) caused pancreatic damage by attenuating oxidative stress, inflammation and apoptosis *via* the Nrf2 signaling pathway (19). Qu et al. found that the mRNA and protein expression levels of Nrf2 and its downstream antioxidant molecules in the male Sprague-Dawley rat spleen were significantly downregulated after supplementing CdCl_2_(20 mg/L) in water for 8 weeks (11). Moreover, in the present research, the inhibited expressions of Nrf2-related molecules (Nrf2, HO-1, NQO-1, SOD, GSH-Px and GST) by Cd were significantly attenuated in male mice administered with NSe. In addition, the NSe group significantly increased expressions of HO-1, GSH-Px and GST compared with the SSe group, indicating that the NSe protective effect is stronger than SSe. Meanwhile, several lines of evidence have suggested that NSe, as a kind of antitoxins, protects organisms from different adverse environmental conditions. For example, Zhang et al. reported that Nano-Se could improve NiSO_4_-induced rat testicular injury *via* regulating the mitochondria-mediated apoptotic pathway (42). In addition, Zheng et al. found that Nano-Se could serve as a novel promising strategy against Hcy-mediated vascular dysfunction (43). Therefore, the positive effects of NSe on poor conditions may be caused by multiple factors, including its stability, small particle size and antioxidant capacity (23). So far, whether NSe could upregulate the inhibition of Nrf2 expression caused by Cd has not been reported, but some of evidence demonstrates that Se can promote Nrf2 expression in tissues, as well as play a cell protective role in the diverse pathological conditions, such as fluorine-induced duodenum and jejunum damage (44). Our results indicated that NSe protects against cadmium-induced acute liver injury in male mice by activating the Nrf2 pathway.

In conclusion, supplement of nano-selenium decreases Cd-induced acute hepatic toxicity through promoting the expression of the Nrf2 pathway related to alleviation of oxidative stress in male Kunming mice. The findings of this study help to better understand the mechanism by which nano-selenium inhibits Cd-induced hepatotoxicity. However, further studies are required to full elucidate detailed mechanism through which nano-selenium acts against Cd-induced hepatotoxicity.

## Data Availability Statement

The datasets presented in this study can be found in online repositories. The names of the repository/repositories and accession number(s) can be found in the article/supplementary material.

## Ethics Statement

The animal study was reviewed and approved by the Institutional Animal Care and Use Review Board of the Sichuan Agricultural University under permit number DYY-2020203012.

## Author Contributions

HD, GS, JL, and HF designed the experiments. YZ, HT, WZ, and FX performed the experiments. LC analyzed the data and wrote the manuscript. BJ, GS, YZ, and FX conducted the final proofreading. All authors have read and agreed to the published version of the manuscript.

## Funding

This work was supported by the National Natural Science Foundation of China (31872347), the Local Science and Technology Development Fund Project Guided by the Central Government of Guizhou Province (20214018), and the Sichuan Science and Technology Innovation Seedling Project Cultivation Project (2021068). All funding bodies provided funding support for the animal purchase, index determination, and open access publication fees.

## Conflict of Interest

The authors declare that the research was conducted in the absence of any commercial or financial relationships that could be construed as a potential conflict of interest.

## Publisher's Note

All claims expressed in this article are solely those of the authors and do not necessarily represent those of their affiliated organizations, or those of the publisher, the editors and the reviewers. Any product that may be evaluated in this article, or claim that may be made by its manufacturer, is not guaranteed or endorsed by the publisher.
